# 2012 Multivariate air pollutant exposure prediction in South Carolina

**DOI:** 10.1017/cts.2018.98

**Published:** 2018-11-21

**Authors:** Ray Boaz, Andrew Lawson, John Pearce

**Affiliations:** Department of Public Health Sciences, Medical University of South Carolina

## Abstract

OBJECTIVES/SPECIFIC AIMS: The objective of this project is the application of complex fusion models, which combine observed and modeled data, to areas with sparse monitoring networks with multiple chemical components is under-developed. Such models could provide improved accuracy and coverage for air quality measurement predictions, an area greatly limited by the amount of missing data. METHODS/STUDY POPULATION: This project focuses on the development of methods for improved estimation of pollutant concentrations when only sparse monitor networks are found. Sparse monitoring networks are defined as areas where fewer than three criteria air pollutants (based on EPA standards) are monitored. Particularly, a multivariate air pollutant statistical model to predict spatio-temporally resolved concentration fields for multiple pollutants simultaneously is developed and evaluated. The multivariate predictions allow monitored pollutants to inform the prediction of nonmonitored pollutants in sparse networks. RESULTS/ANTICIPATED RESULTS: Daily, ZIP code level pollutant concentration estimates will be provided for 8 pollutants across South Carolina, and goodness of fit metrics for model variants and previously established methods will be compared. DISCUSSION/SIGNIFICANCE OF IMPACT: These methods utilize only widely available data resources, meaning that the improved predictive accuracy of sparsely monitored pollutant concentrations can benefit future studies in any US area by improving estimation of health effects and saving resources needed for supplemental air pollutant monitoring campaigns. Our method for estimation attempts to improve predictive accuracy and data availability for sparsely monitored pollutants and areas.

